# Negative Neuroplasticity in Chronic Traumatic Brain Injury and Implications for Neurorehabilitation

**DOI:** 10.1007/s11065-014-9273-6

**Published:** 2014-11-25

**Authors:** Jennifer C. Tomaszczyk, Nathaniel L. Green, Diana Frasca, Brenda Colella, Gary R. Turner, Bruce K. Christensen, Robin E. A. Green

**Affiliations:** 1 Research Department, Toronto Rehabilitation Institute - University Health Network, Toronto, ON Canada; 2 Faculty of Arts and Science, University of Toronto, Toronto, ON Canada; 3 Department of Psychiatry, Division of Neurosciences, University of Toronto, Toronto, ON Canada; 4 Department of Psychology, York University, Toronto, ON Canada; 5 Department of Psychiatry and Behavioural Neurosciences, McMaster University, Hamilton, ON Canada; 6 Department of Psychiatry, University of Toronto, Toronto, Canada; 7 Department of Rehabilitation Science, University of Toronto, Toronto, ON, Canada; 8 Present Address: Faculty of Medicine, Technion Israel Institute of Technology, Haifa, Israel; 9 Present Address: Research School of Psychology, Australian National University, Canberra, Australia

**Keywords:** Traumatic brain injury, Negative neuroplasticity, Post-acute decline, Adult, Cognitive aging, Moderate to severe traumatic brain injury

## Abstract

Based on growing findings of brain volume loss and deleterious white matter alterations during the chronic stages of injury, researchers posit that moderate-severe traumatic brain injury (TBI) may act to “age” the brain by reducing reserve capacity and inducing neurodegeneration. Evidence that these changes correlate with poorer cognitive and functional outcomes corroborates this progressive characterization of chronic TBI. Borrowing from a framework developed to explain cognitive aging (Mahncke et al., *Progress in Brain Research, 157*, 81–109, 2006a; Mahncke et al., *Proceedings of the National Academy of Sciences of the United States of America, 103*(33), 12523–12528, 2006b), we suggest here that environmental factors (specifically environmental impoverishment and cognitive disuse) contribute to a downward spiral of negative neuroplastic change that may modulate the brain changes described above. In this context, we review new literature supporting the original aging framework, and its extrapolation to chronic TBI. We conclude that negative neuroplasticity may be one of the mechanisms underlying cognitive and neural decline in chronic TBI, but that there are a number of points of intervention that would permit mitigation of this decline and better long-term clinical outcomes.

Traumatic brain injury (TBI) can severely disrupt the cognitive, social, emotional and physical functioning of affected individuals. These negative consequences exact a further toll on friends, family, colleagues and community members (Andelic et al. 2010; Hawthorne et al. 2009; Resch et al. 2009). TBI also causes a significant financial burden for society more broadly (McGarry et al. 2002; Polinder et al. 2005). Given that TBI affects an estimated 10 million people worldwide each year (Hyder et al. 2007), and that many people living with TBI—especially moderate to severe TBI—require rehabilitation services for an extended period of time (National Institutes of Health 1998), research and clinical efforts to improve recovery from TBI are imperative.

Traditionally, TBI has been conceptualized as a discrete event—an injury—followed by a protracted, but circumscribed, recovery period (Masel and DeWitt 2010; Ng et al. 2008). This recovery period has been conventionally characterized by gradual gains in functioning, an eventual plateau at a level that is less than that of pre-injury functioning (Christensen et al. 2008; Povlishock and Katz 2005), and maintenance of gains thereafter. However, there is mounting evidence that this view of recovery does not fully capture the long term trajectory of TBI, and in particular the chronic effects (Till et al. 2008; Millis et al. 2001; Himanen et al. 2006). For some TBI patients, the above-noted plateau in functioning is followed by cognitive *decline* during the early and later chronic stages of injury (Hammond et al. 2004; Masel and DeWitt 2010; Millis et al. 2001; Ruff et al. 1991; Till et al. 2008; Himanen et al. 2006). In a study by Ruff et al. (1991), one third of patients in their sample showed a 20 % or greater drop in verbal learning from 6 to 12 months post-injury. In a study by our group conducted from 1 to 2 years post-injury (Till et al. 2008), just over 25 % of patients showed substantive decline in two or more domains of cognitive function; and, in a study by Himanen et al. (2006), 56 % of patients showed cognitive decline from approximately 2.5 to 30 years post-injury.

Substantive neural losses in the chronic stages of injury are observed as well. Our group has shown lesion expansion, atrophy of the whole brain and hippocampi, and loss of white matter integrity from approximately 4.5 months to 2.5 years post-injury (Adnan et al. 2013; Greenberg et al. 2008; Ng et al. 2008). (Importantly, the initial time point of these studies was late enough that changes were unlikely to be attributable to the resolution of acute neurological events such as resolution of edema and gliosis.) A number of other groups have observed similar such structural losses across time (Farbota et al. 2012a, b; Sidaros et al. 2009; Johnson et al. 2013).

A small number of studies have found a link between volumetric change and behavioural change (Bigler et al. 1997; Blatter et al. 1997). For example, Farbota et al. (2012a) used diffusion tensor imaging (DTI) to examine changes in white matter integrity in a small sample of chronic stage TBI patients over a 4-year post-injury period; over this time span, TBI patients demonstrated fractional anisotropy (FA) reductions in the corpus callosum that were associated with poorer performance on a test of manual motor functioning (i.e., finger tapping).

Finally, our group has demonstrated that structural losses in the chronic stages of injury are ubiquitous (Green et al. 2014). In a sample of 56 patients with complex-mild to severe TBI, 54 showed significant volume losses as compared to controls in the whole brain, right or left hippocampus or corpus callosum, with the majority showing losses on three of these four measures.

Encouragingly, there is some suggestion that the mechanisms underlying these deleterious changes may be modifiable factors that occur *post*-injury. In the above study, the very high prevalence of significant decline in our heterogeneous sample (96 %) implicates post-injury factors rather than pre-morbid risk factors (e.g., genetic pre-disposition), which would be present in only a subset. Bolstering this interpretation was our finding in a separate study of these patients that degree of self-reported environmental enrichment (cognitive stimulation in particular) at 5 months post-injury was negatively associated with degree of bilateral hippocampal volume atrophy from 5 to 28 months post-injury (Miller et al. 2013), a finding offering the possibility that post-injury environmental factors might mitigate volumetric losses.

Post-TBI neurophysiological mechanisms that have been implicated in neurodegeneration include protein deposition (e.g., amyloid and tau) and neuroinflammation (Bigler 2013a, b; Smith et al. 2013; Johnson et al. 2013). Johnson et al. (2013) found evidence of increased neuroinflammation (dense regions of reactive microglia) in the corpus callosum of TBI patients in the sub-acute and chronic stages of injury as compared to healthy controls, as well as reduced thickness of this structure. Several other studies have found associations between neuroinflammation and chronic stage cognitive impairments (for a review see Kumar and Loane 2012; Ramlackhansingh et al. 2011). It is critical to underscore that while these post-injury factors are currently compromising long-term outcomes, they also open new avenues for intervention, and thus offer new hope for improving long-term outcomes.

Despite the growing awareness of the chronic and apparently progressive nature of TBI (Ng et al. 2008; Till et al. 2008; Green et al. 2014; Adnan et al. 2013), there has yet to be a neurorehabilitation framework that characterizes this downward course of cognitive and brain function in the chronic stages of TBI and the factors that contribute to decline. Such a framework could serve several purposes: to highlight points of intervention for improving recovery, to develop testable hypotheses for future research, and to disseminate the reconceptualization of moderate-severe TBI as a deteriorative disorder.

To this end, we extrapolate from the “negative plasticity” framework of Mahncke et al. (2006a) that was proposed to explain cognitive decline in older adults. As described in greater detail later, their framework describes a self-reinforcing, downward spiral of negative brain plasticity whereby declining brain function is attributable to a combination of disuse (called “reduced schedules of activity”), reduced quality of sensory-perceptual processing, and weakened neuromodulatory control. In combination, these factors increase reliance on simplified cognitive processing at the expense of more complex processing capacity (called “negative learning”). These processing changes result in brain changes, which in turn result in further disuse, perceptual compromise and reduced neuromodulatory control.

We suggest that chronic stage TBI patients undergo a similar downward spiral for related reasons: Initial impairments to primary cognitive, sensory-perceptual and neuromodulatory function, secondary to TBI, lead to reductions in functional capacities (e.g., the ability to communicate or to regulate emotions in professional or social situations, the ability to remember to a conversation). This results in reduced participation in activities that demand complex cognitive processing, such as academic, occupational and social activities. These factors, in combination, may foster brain adaptations to simpler and more habitual cognitive processes at the expense of complex processing and underlying neural functioning. Following the framework of Mahncke et al. (2006a), we suggest that the brain is thereby at risk of disuse-mediated negative neuroplastic change, thereby leading to some of the progressive deterioration that is observed in chronic TBI.

The older adult framework extrapolates easily to chronic TBI because of numerous parallels between the populations. Older adults undergo a reduction in their schedules of activity (or participation) due to retirement and loss of social networks. Patients in the chronic stages of TBI similarly undergo a reduction in participation after the most intensive of their therapies have concluded, but patients have not returned to an active occupational, school or social life (Frasca et al. 2013). Older adults undergo aging-related sensory-perceptual and cognitive impairments as well as diminished neuromodulatory control. Many chronic moderate-severe TBI patients suffer persisting sensory-perceptual decrements (e.g., Greenwald et al. 2012), altered neuromodulatory functioning (e.g., Bales et al. 2009) and cognitive impairment as a result of structural and functional damage to the brain (e.g., Bashore and Ridderinkhof 2002; Anderson and Knight 2010; Arenth et al. 2012; Christensen et al. 2008). Lastly, there is overlap in the nature of brain changes secondary to aging and to moderate-severe TBI such as structural and functional alterations to the temporal and frontal lobes (Bigler and Maxwell 2011), reduced white matter integrity (Arenth et al. 2013), and brain volume loss (Farbota et al. 2012b; Ross 2011; Sidaros et al. 2009).

The idea that chronic stage neurodegeneration in TBI patients may be caused by disuse-related negative plastic changes converges with the large body of animal research into environmental enrichment (EE). EE is typically operationalized as an environment that provides augmented opportunity for intensified cognitive stimulation, as well as physical exercise and social stimulation. A seminal study on this topic was undertaken by Hebb (1947), who reared rats in his family home as pets and compared their cognitive outcomes to rats reared in cages. He found that the pet rats exhibited superior problem-solving abilities to the caged rats. Since then, a wealth of studies has demonstrated the beneficial effects of EE on cognitive performance and brain function, and relevant to the arguments here, the detrimental effects of impoverished environments (Bernstein 1972; Winocur 1998; for reviews see Simpson and Kelly 2011; Petrosini et al. 2009). The EE literature shows the critical relevance of cognitive stimulation for cognitive functioning in animals, at the level of the neuron, for example influencing adult neurogenesis and synaptic plasticity (for a review see Hannan 2014; Rosenzweig 1966; Rosenzweig and Bennett 1996; Van Praag et al. 2000) and even in mitigating aspects of neurodegeneration (e.g., delaying onset, slowing progression, or remediating neural pathology) in rodent models of Alzheimer’s, Parkinson’s and Huntington’s disease (for a review see Hannan 2014).

The potential to reverse the deleterious brain and behavioural changes brought about by disuse is critical to the current framework’s use in a neurorehabilitation context. It is encouraging that a number of studies demonstrate the reversibility of the effects of impoverished environments when animals are later exposed to enriched environments (Kobayashi et al. 2002; Lu et al. 2003) in both younger and older (Speisman et al. 2013; Winocur 1998) animals as well as in animals who have sustained a TBI (for a review see Frasca et al. 2013). For example, Kobayashi et al. (2002) found that juvenile rats raised in impoverished conditions that were subsequently exposed to 3 months of enrichment as adults showed better problem-solving than their counterparts that were maintained solely under the impoverished conditions. Also, Lu et al. (2003) found that juvenile rats reared in isolation for 4 weeks, and then reared in group housing for the subsequent 4 weeks, showed a reversal of the deleterious effects on water maze task performance, and long-term potentiation of CA1 neurons.

Other EE literature with direct relevance to the current framework discusses findings on TBI. For example, animals exposed to EE following injury show relative improvements in motor and cognitive function, and these changes are associated with changes in neural function such as less cell body degeneration, improved neurogenesis, as well as reduced lesion size (for a review see Bondi et al. 2014; Hamm et al. 1996; Johansson and Ohlsson 1996). Conversely, the animals not exposed to this stimulation show a much poorer course of cognitive, motor and neural recovery.

The overall objectives of this paper are twofold. The first is to provide an updated and expanded review of the literature that supports the negative neuroplasticity framework in the original context of human aging. The original framework focused in large part on animal literature; however, newer literature provides further support from human studies. The second objective is to extrapolate this framework to a discussion of chronic TBI and neurodegeneration. Here, we adapt the Mahncke et al. (2006a) framework for use with the chronic moderate-severe TBI population, and discuss the possibility of negative neuroplasticity as a putative mechanism of cognitive and neural decline in chronic TBI. We also discuss some of the implications for neurorehabilitation.

## Negative Neuroplasticity in Aging

Cognitive and neural decline in aging have been well-established (Hedman et al. 2012; Salthouse 2010). Cognitive changes include declines in episodic memory (Craik and Rose 2012), selective attention and inhibition of distracting information (Campbell et al. 2012; Cashdollar et al. 2013; McGinnis 2012), speed of processing (Salthouse 2000), and executive functions, including task switching, planning, decision making, verbal fluency and working memory (Gold et al. 2013; Kavé and Mashal 2012; Köstering et al. 2013; Laguë-Beauvais et al. 2013; Luo et al. 2013; Yoon et al. 2009). This aging-related waning of cognitive faculties has been associated with specific brain changes. The frontal and temporal lobes are particularly vulnerable to atrophy, especially medial temporal areas and the hippocampi (Gonoi et al. 2010; Raz et al. 2010). White matter integrity also decreases during aging, (Burzynska et al. 2010), and disruptions of neural networks may play a role in aging-related cognitive impairments (Campbell et al. 2013). Reductions have been observed in number of dendrites, dendritic spines, and synapses, particularly in the prefrontal cortex (Pannese 2011; Peters and Kemper 2012) and in medial temporal lobe structures (Morrison and Baxter 2012). Aging-related changes in head volume of thin spines on dendrites have been associated with worse cognitive performance (Dumitriu et al. 2010), and hippocampal volume changes have been associated with reduced levels of brain-derived neurotrophic factor (BDNF; Erickson et al. 2010).

As noted above, Mahncke and colleagues (Mahncke et al. 2006a; Mahncke et al. 2006b) have argued that a mediator of the cognitive and physiological aging described above is negative neuroplastic change (Vance et al. 2010). Here, increased reliance on simpler processing strengthens underlying neural pathways in a Hebbian Learning manner (i.e., “the cells that fire together wire together”; Hebb 1949) with commensurate decreases in the activity of competing, complex cognitive processes, resulting in a weakening of their underlying synaptic connectivity (Mahncke et al. 2006a; Dosher and Lu 2009). They thus conceptualize negative plastic change as “a self-reinforcing, downward spiral of reduced interaction with challenging environments and reduced brain health” (Mahncke et al. 2006a, p.86). Mahncke and colleagues (Mahncke et al. 2006a, b) proposed that three inter-related factors contribute to these negative neuroplastic changes: (1) reduced schedules of activity (or disuse), (2) noisy processing, and (3) weakened neuromodulatory control. These factors combine to produce negative learning. Negative learning, a behavioural phenomenon (i.e., the reliance on simplified processing at the expense of more complex processing), gives rise to negative neuroplastic change, the neurophysiological underpinning of cognitive decline.

Here, we provide updated evidence for each of the factors from the negative neuroplasticity framework alongside their explication, in order to provide an empirical basis for later discussing these factors in the context of our adaptation of the framework for chronic TBI. We note that although a number of other factors may possibly contribute to aging- and TBI-related cognitive and neural decline through negative neuroplastic brain changes, Mahncke et al. (2006a) focus on those factors that may be readily modifiable through behavioural interventions.

### Reduced Schedules of Activity

Secondary to a range of environmental and subject factors (e.g., retirement, shrinking social networks due to morbidity and mortality), older adults typically experience *reduced schedules of activity* (Nimrod et al. 2009; Pushkar et al. 2010), including (but not limited to) restricted physical/motor activity, and mobility restrictions (Buchman et al. 2009), driving cessation (Edwards et al. 2009; Mezuk and Rebok 2008), and attenuated variety/novelty seeking activities (Novak and Mather 2007; Bouisson 2002). Moreover, research indicates that activity reductions may be purposefully chosen by older adults (Bouisson and Swendsen 2003); that is, given the freedom to schedule their daily activities, older adults may avoid novel and/or challenging activities. For example, older adults have been found to have lower rates of daily social contact compared to young adults (Cornwell 2011), and work examining social behaviour has demonstrated older adults’ preference for familiar over novel social partners (Fung and Carstensen 2006). Such alterations in behaviour, in particular social withdrawal, might logically be influenced by other factors in the current framework, such as reduced reward (secondary to reduced neuromodulatory control) or reduced perceptual fidelity (Evans et al. 2008).

The contention that reduced schedules of activity is associated with poorer cognitive aging is supported by Winocur and Moscovitch (1990), who showed that older adults who were community-dwelling (which we equate to a complex environment) had better cognitive function than institutionalized older adults (which we equate to a relatively impoverished environment), even after controlling for differences in general intelligence, age, and health. Similarly, studies have shown that less socializing is associated with greater aging-related cognitive decline (e.g., James et al. 2011), including one study that found that older adults who are socially vulnerable (low levels of social activity and social support) are 36 % more likely to experience declines in global cognitive function over a 5 year period than older adults with low social vulnerability (Andrew and Rockwood 2010).

Relevant to the current paper is the growing number of “use it or lose it” studies demonstrating a positive association between level and/or variety of cognitive activities and cognitive function. For example, Eskes et al. (2010) found that diversity of cognitive activities in older women was associated with better cognitive function, and Small et al. (2012) found declines in frequency of cognitive activities to be associated with declines in memory and speed-of-processing. Moreover, Wang et al. (2013) found similar associations between the frequency of cognitive, social, and physical activities and cognitive function in a sample of predominately illiterate Chinese older adults. Interestingly, these researchers found a dose-dependent relationship between activity frequency and cognitive decline: engagement in higher levels of at least one activity type was related to stability or improvement in cognitive function, whereas low levels of activity participation were associated with cognitive decline. Although most studies in this area are correlational and observational in design, one recent study experimentally manipulated level of cognitive activity. In this study, older adults who learned complex tasks (quilting, digital photography) over 3 months of intensive study (about 16 h/week) demonstrated superior episodic memory compared to older adults who participated in social activities or less complex cognitive tasks (Park et al. 2014). Further prospective studies in this area are needed to verify that increased stimulation causes increased cognitive function, and to rule out the alternative explanation that people with higher cognitive functioning engage in more cognitively stimulating activities.

### Noisy Processing

The negative neuroplasticity framework speculates that aging-related sensory degradation results in disproportionately *noisy processing*. These changes make it more difficult to discern signals from internal noise, leading to reductions in speed and the quality of mental processing (Humes et al. 2013). In older adults, peripheral nervous system changes to vision (e.g., decreases in visual acuity, low-light accommodation, colour vision) (Dorey and Blanks 2009) and hearing (e.g., decreased pitch perception, sound encoding precision) (Clinard et al. 2010; Russo et al. 2012) are believed to underpin declines in brain function because sensory brain areas are activated less frequently and/or less robustly (Cliff et al. 2013). Poorer hearing ability in older adults has been associated with decreased activation in the primary auditory cortex during reading of grammatically complex sentences, and with smaller auditory cortex volume (Peelle et al. 2011). Such auditory declines have also been associated with older adults’ ability to cognitively process information (Humes et al. 2013; McDaniel et al. 2008). A recent study estimated that a mild decline in hearing ability is equivalent to an increase of 6.8 years in chronological age in terms of the negative effects on cognitive function (Lin et al. 2011). Similarly, studies have found that poorer visual function in older adults is related to worse fluid intelligence and memory span, (Clay et al. 2009), and to diminished episodic memory, reasoning, and speed of processing (Weatherbee et al. 2009). Thus, it appears that an important consequence of degradations in sensory processing is a robust compromise in cognitive processing. Critically, according to the Mahncke et al. (2006a) framework, sensory-perceptual impairments lead to consequent reductions in functional communication. These in turn result in reduced schedules of activity, with individuals avoiding complex or challenging interactions due to their compromised functioning.

### Weakened Neuromodulatory Control

A third factor contributing to negative neuroplastic change put forward by Mahncke and colleagues (Mahncke et al. 2006a) is *weakened neuromodulatory control*. After Rostène et al. (2007), we use the term neuromodulator to refer to “a neurotransmitter that by itself has no effect under basal conditions but that modulates induced neuronal activity” (Rostène et al. 2007, p. 901). Our focus, as in Mahncke et al. (2006a), is on the neuromodulators involved in neuroplastic changes that occur with learning (e.g., acetylcholine, dopamine, serotonin, and norepinephrine). Mahncke et al. (2006a) posit that aging-related changes in several neurotransmitters implicated in neuromodulatory control result in negative plasticity and, thus, cognitive decline. Two of the chief neuromodulatory systems implicated in the process of aging-related negative neuroplasticity are dopamine and acetylcholine. Dopamine (DA) is known to be involved in response to reward and novelty during learning (Krebs et al. 2011). It is well established that the prevalence of DA in the brain decreases with age, and research has demonstrated that this decrease is related to poorer cognitive function, particularly executive function (Bäckman et al. 2010). For example, older adults who successfully learned to overcome response conflicts to obtain a monetary reward in a go/no-go task had greater structural integrity of the substantia nigra and ventral tegmental area than those who were unsuccessful (Chowdhury et al. 2013b). In addition, older adults with greater structural connectivity between these areas and the striatum performed just as well as younger adults on probabilistic learning tasks, and poor performance among older adults was boosted by administration of the DA precursor, L-DOPA (Chowdhury et al. 2013a). What is more, aging-related decreases in DA levels underlie the decreased recruitment of task-relevant brain areas seen in older compared to younger adults for executive functions such as working memory (Bäckman et al. 2011).

Acetylcholine is intimately involved in attention, learning and memory, and is also known to undergo aging-related changes as a result of degeneration of cholinergic neurons (Klinkenberg et al. 2011; Störmer et al. 2012). Research shows that less integrity of the basal forebrain (which contains a large concentration of cholinergic neurons that project to the cortex), as assessed by magnetization transfer ratio, is related to poorer performance on long term and working memory tests in older adults (Düzel et al. 2010). Also, lower levels of cholinergic activity were found to be related to poorer performance on tests of episodic memory in a sample of younger and older adults (Young-Bernier et al. 2012).

Although there is currently no direct evidence of a causal link between weakened neuromodulatory control in older adults and negative neuroplastic change, there exists indirect evidence from animal research that suggests a possible causal role for neuromodulatory function in the control of subjects’ engagement in activities. Anhedonia, defined as “loss of interest or pleasure in all, or almost all, activities” (Der-Avakian and Markou 2012 p. 68), typically seen in major depressive disorder and schizophrenia, can be thought of as a kind of disengagement from activities, including cognitive activities. A number of neurotransmitters are known to regulate anhedonia, and as such may regulate engagement (cognitive and otherwise) with the environment. For instance, the activation of μ opioid, endocannabinoid, and GABA_A_ receptors in the nucleus accumbens and ventral pallidum results in less anhedonic behaviour in rodents (Der-Avakian and Markou 2012). Also, knockout mice missing genes for these neurotransmitters also show greater anhedonic behaviour (i.e., less preference for sucrose compared to water, in sucrose intake or preference tests) (Der-Avakian and Markou 2012). Thus, these neurotransmitters may play a role in modulating levels of engagement in the environment and so contribute to disengagement and, ultimately, negative neuroplasticity.

### Negative Learning

*Negative learning* is theoretically postulated to occur when, as a result of reduced schedules of activity, noisy processing, and weakened neuromodulatory control, an individual relies on more familiar and habitual routines and simpler and less effortful cognitive processes (Mahncke et al. 2006a). Such an approach serves to further accelerate cognitive declines in a self-reinforcing manner (Mahncke et al. 2006a). For example, a hypothetical retired older adult who is less inclined to participate in challenging cognitive activities compared to when he was working, may also experience problems with his hearing, as well as memory and learning ability due to alterations in acetylcholine function. These changes may make him less likely to decide to take on new learning during his retirment (e.g., a course to learn a new language), and this reduced engagement would in turn permit or induce him to rely on simpler, more heuristic cognitive processes. In this scenario, negative learning would contribute to disuse of brain areas involved in more complex cognitive operations (e.g., using frontal lobe-based mnemonic strategies), or negative neuroplasticity.

Increased reliance on the simpler or at least less effortful cognitive processing that is associated with negative learning in this framework is observed in several lines of research in older adults. Older adults show reduced semantic clustering (retrieval of groups of semantically related words) in free recall tasks compared to younger adults (Kirchhoff et al. 2014; Taconnat et al. 2009). As self-initiated use of this elaborative recall strategy is related to intact frontal lobe function, it is considered a more complex and effortful strategy than others such as serial clustering (Kirchhoff et al. 2014). Older adults also have difficulty using detailed, recollective memory as compared with familiarity-based memory to make recognition judgments (Edmonds et al. 2012), especially when task conditions are made more difficult (Angel et al. 2013). Moreover, false memories (to which older adults are more susceptible than young adults) can result from the greater influence of automatic processing and familiar stimuli (Jacoby and Rhodes 2006). Some studies suggest that older adults also have difficulty correctly recognizing novel pairings of familiar stimuli than younger adults, indicating that older adults may have difficulty overriding the familiarity of the individual stimuli (Kersten and Earles 2010).

As well, older adults perform better on cognitive tasks for which they can rely on environmental support such as pre-existing representations in memory and previously acquired knowledge. For instance, when learning the value of one stimulus in relation to other stimuli, such as in a game similar to *rock-paper-scissors* in which stimuli are novel objects, older adults are just as accurate in learning these relations as younger adults if they can first practice the task with familiar stimuli for which the value of stimulus relations are known. This suggests that older adults benefit from associating novel tasks with previous knowledge (Ostreicher et al. 2010).

Furthermore, a number of studies show that older adults are more likely to employ less cognitively demanding strategies to complete tasks compared to their younger counterparts (Rodgers et al. 2012; Taconnat et al. 2009). For example, Bohbot et al. (2012) examined use of different navigational strategies in a virtual maze in a cross-sectional sample of children, younger adults, and older adults. Whereas the use of a spatial strategy (using the relative positions of landmarks for way finding, known to activate the hippocampus) was more prevalent in children and younger adults, the use of a response strategy (memorizing a series of left and right turns relative to starting location, known to activate the caudate nucleus) was more prevalent in older adults. Given the caudate’s role in more automatic processing, Bohbot et al. (2012) hypothesized that the use of (caudate-based) response strategies in older adults may represent a bias toward more routinized, automated behaviour, perhaps as a result of aging-related hippocampal degradation. Moreover, older adults are less likely to employ memory strategies that require self-initiated processing compared with younger adults, favouring the use of external (e.g., calendars) over internal mnemonic strategies (e.g., visual imagery) in their everyday lives (Bouazzaoui et al. 2010). The less frequent use of internal memory strategies in older adults has been associated with poorer executive function, suggesting that external memory strategies may compensate for aging-related reductions in executive function (Bouazzaoui et al. 2010). Aging-related differences in strategy use have also been found in the context of decision making; older adults have been shown to make worse decisions when they must use a cognitively effortful strategy to optimize their decision making, compared to when they must use a simpler strategy (Mata et al. 2010).

Taken together, findings on aging-related changes in cognitive processing and strategy use suggest that older adults rely less on effortful cognitive processing. The many examples of increased reliance on familiar, routine and less effortful processing described above support a relationship between aging and negative learning. The causes of such shifts in processing are likely multi-factorial, and further research is needed to identify whether such shifts can be causally attributed to any or all of the factors described in the negative neuroplasticity framework, namely reduced schedules of activity, noisy processing and reduced neuromodulatory control.

### Direction and Causality of Relationships

Increased reliance on routine, less effortful, familiar processing would logically lead to less activation of brain networks devoted to effortful processing, an example of negative neuroplasticity that might explain the neural decline characteristic of aging. This idea is consistent with the above-mentioned findings that older adults who engage in only low levels of activities show greater cognitive decline, whereas those who engage in high levels of activity show cognitive stability or improvement (Wang et al. 2013). In the original framework, Mahncke et al. (2006a) contend that the downward spiral of negative neuroplastic change may be originally caused by behavioural change that initiates reduced brain activity or by reduced brain function due to aging-related physiological changes, or by both. Ascertaining the starting point for the downward spiral is not relevant to their framework because it is the aggregate of the factors, and the dynamic relationship between them, that gives rise to negative plasticity.

Nonetheless, with regard to the relationship between the factors of the framework, further research is needed. A number of lines of evidence support the existence of each of the factors of the negative neuroplasticity framework in the context of cognitive aging, but relationships between the factors have limited empirical support and there are several gaps for which further research is needed. For example, is the relationship between neuromodulatory control, reduced schedules of activity, and noisy processing additive or synergistic? Does a causal relationship exist between each of these factors and negative learning? What is the evidence that negative learning induces changes to brain function at the level of higher cognitive functions (as opposed to a sensory-perceptual level)? Lastly, what is the empirical evidence that negative learning causes negative neuroplastic change and associated declines in brain health? Thus, a helpful next step in empirically validating the framework would be to employ prospective, longitudinal experimental designs with behavioural and imaging outcomes (including structural and functional connectivity measures) to clarify causal relationships between components of their framework.

**Fig. 1:**
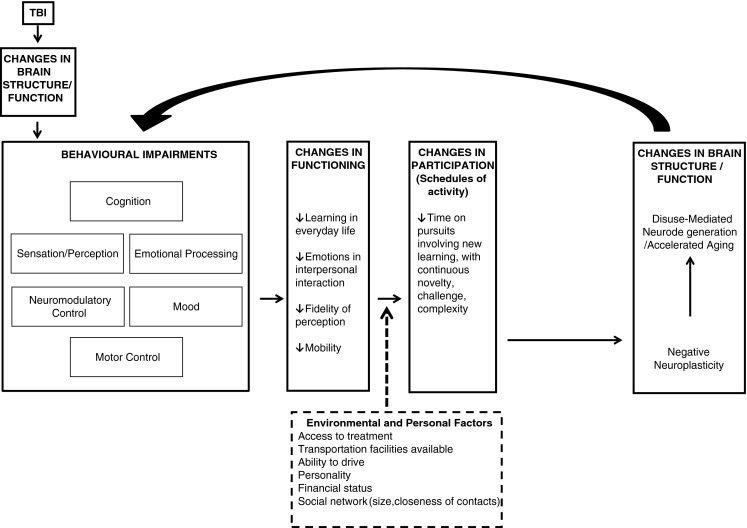
Depiction of framework in context of TBI, showing neurodegeneration in chronic TBI secondary to negative learning/neuroplasticity. Figure adapted from Mahncke et al. (2006a), employing elements of the International Classification of Functioning, Disability and Health framework (World Health Organization 2013). *Arrows* between boxes (and within the “Changes in Brain Structure/Function” box) indicate postulated direction of causal influence, with *solid arrows* indicating direct influence and *dashed arrow* indicating modulator variables. In this adapted framework, various behavioural impairments caused by TBI result in deleterious changes in functioning and participation. As a result, negative learning creates negative neuroplastic brain changes resulting in neurodegeneration, which feed back to worsen behavioural impairments. Environmental and personal factors are posited to modulate the impact of changes in functioning on participation

In our extrapolation of the Mahncke et al. (2006a) framework to chronic TBI, discussed in the next section, we argue for a number of specific causal relationships between components of the framework and a clear point at which changes are initiated (See Fig. 1). We present the available evidence to argue that an initial decline in brain structure and function caused by the TBI initiates the behavioural impairments that lead to deleterious functional changes. Such losses reduce the patient’s motivation or capacity to participate in cognitively demanding activities, resulting in disuse (or negative learning). This in turn gives rise to the downward spiral of negative neuroplastic change that causes some of the disuse-mediated neurodegeneration observed in chronic TBI.

## Utility of the Negative Neuroplasticity Framework for Understanding Neurodegeneration in TBI

We propose that the negative neuroplasticity framework may be applicable to understanding the effects of chronic stage moderate to severe TBI. Notably, each of the factors outlined in relation to aging are also present in the TBI population, as outlined in our previous paper (Evans et al. 2008). Moreover, despite obvious differences between the two populations (e.g., the precipitating mechanism of brain changes, severity of impairments) there is substantial overlap in the kinds of behavioural (i.e., sensory-perceptual and cognitive) and neurophysiological alterations observed in older adults and those observed in moderate to severe TBI patients. As in aging, common cognitive deficits include impairments to attention (Mangels et al. 2002), speed of processing (Bashore and Ridderinkhof 2002), episodic memory (Arenth et al. 2012; Palacios et al. 2013), and executive functions (Anderson and Knight 2010; Kasahara et al. 2011; Kim et al. 2012; Leunissen et al. 2013; Ord et al. 2010; Slovarp et al. 2012; Sozda et al. 2011), including decision making (Wood and McHugh 2013) and problem-solving (Cazalis et al. 2006). At the level of the brain, damage to temporal and frontal lobes is common (for a review see Bigler and Maxwell 2011), as well as reduced integrity of white matter structures such as the corpus callosum (Arenth et al. 2013). Also, TBI patients experience loss of total brain volume and widespread discrete volume losses in cortical and subcortical areas, (Farbota et al. 2012b; Ross 2011; Sidaros et al. 2009). As with older adults (Cabeza 2002), TBI patients also experience reductions in functional asymmetries during cognitive tasks (Turner et al. 2011). At the neural level, TBI is associated with death of mature neurons in the dentate gyrus of the hippocampus as well as degeneration of dendritic spines and branches, and synapses (Gao et al. 2011; Scheff et al. 2005). Many of these changes underlie impairments in memory and learning after TBI (Gao et al. 2011).

Similar to aging, TBI patients show losses to sensory-perceptual functioning (Greenwald et al. 2012), and in the chronic stages of injury, extended, if not permanent, reductions in EE (e.g., loss of therapies without resumption of employment and/or leisure activities) and consequent reductions in cognitive stimulation (Frasca et al. 2013).

The interest in extrapolating this framework to TBI stems from the growing evidence for decline in the chronic stages of injury, the causes of which may be related to the causes of decline in aging. In this section we discuss evidence for Mahncke and colleagues’ (Mahncke et al. 2006a) factors that are purported to precipitate negative neuroplasticity and hence neurodegeneration in the chronic stage of TBI. We couch this discussion in a preliminary version of a framework that we have adapted slightly from the original, for use with the chronic TBI population (see Fig. 1). The revisions take into consideration the Mahncke et al. (2006a) factors in the context of other factors relevant to TBI. As such, we view the Mahncke et al. (2006a) factors as a part of a more expansive picture of the variables that lead to negative neuroplasticity and neurodegeneration in chronic moderate-severe TBI. We focus on the Mahncke et al. (2006a) factors, however, as they target processes that are likely to be modifiable through intervention. We have also incorporated components of the International Classification of Functioning, Disability and Health (World Health Organization 2013) framework for ease of comparison with other studies.

### Reduced Schedules of Activity: Disuse

TBI patients and their caregivers often report that the transition from rehabilitation to home is characterized by increased idle time, boredom, and little-to-no engagement in meaningful activities (Turner et al. 2009; Turner et al. 2007; Frasca et al. 2013). Moreover, TBI survivors experience disconnection from former professional and social networks (for a review in TBI see Morton and Wehman 1995) thereby affecting vocational and social schedules of activity. TBI patients are often less socially engaged, feel socially isolated and lonely, and may not be able to resume their previously challenging work activities (Bulinski 2010; Morton and Wehman 1995) forcing a complete retirement or a drastic reduction from their pre-injury type and level of employment (Green et al. 2008).

Declines in community integration are also common. One study tracked scores on the Community Integration Questionnaire over a 36 month post-injury period in moderate to severe patients. Although home integration returned to pre-injury levels, both social integration and productivity remained lower than pre-injury levels (Willemse-van Son et al. 2009). Moreover, low-income individuals were found to be especially susceptible to poor social integration after TBI (Sander et al. 2009), and physical barriers (e.g., accessibility) and poor communication skills served to compound already reduced community and social integration (Fleming et al. 2013; Struchen et al. 2011). In addition, participation in leisure activities is found to be reduced after TBI, and with associated increases in television watching (Bier et al. 2009). One of the causes cited for this pattern is difficulty securing transportation, which is a known barrier to leisure participation (Bier et al. 2009). Wise et al. (2010) reported that after 1 year post-injury, 81 % of TBI patients had reduced frequency of participation in leisure activities relative to pre-injury levels. Patients were most likely to experience reductions in the frequency with which they attended social activities and participated in sports, whereas television watching was most likely to be added to their repertoire of activities.

Reduced schedules of activity in moderate to severe TBI patients have been shown to be associated with poorer neural outcomes. For example, a study in our lab (Miller et al. 2013) found a negative correlation between TBI patients’ level of self-reported daily activities (indicative of EE) at 5 months post-injury and atrophy of the hippocampi bilaterally from 5 to 28 months post-injury.

Hence, a number of lines of evidence support the contention that TBI patients experience reduced schedules of activity. Moreover, there is some early evidence that reduced schedules of activity may contribute to the negative neuroplasticity that gives rise to observed atrophy in chronic TBI. Importantly, one of the environmental factors cited as a barrier to participation (and hence a potential moderator of negative learning)—difficulty securing transportation—represents a potentially modifiable factor.

### Noisy Processing

TBI patients can experience simultaneous deficits in both vision and hearing (Lew et al. 2010). A recent review by Greenwald et al. (2012) reported that blurred vision, double vision, visual field defects, reading difficulties, light sensitivity, and colour blindness were common visual impairments caused by TBI, and traumatic photosensitivity has been observed even in mild TBI populations recently, with early evidence of prevalent and enduring symptoms (Truong et al. 2014; Capó-Aponte et al. 2012; Goodrich et al. 2013) self-reported after 1 year post-injury. TBI patients may also experience deficits in hearing (Koshimori et al. 2009), with magnitude of deficits positively correlating with injury severity, particularly for high frequency sounds (Munjal et al. 2010). One study of veterans who had sustained (non-blast induced) TBI found that 44 % of patients self-reported hearing loss and 18 % complained of tinnitus (Lew et al. 2007).

Recent research has begun to demonstrate a link between changes in hearing and changes in the brain. For instance, Tate et al. (2014) found evidence of cortical thinning in the left superior temporal and frontal gyri in service members who had sustained a blast-induced mild TBI, compared to age- and gender-matched service members who had also been deployed; service members with blast-induced TBI were also found to have high rates of hearing disorders such as tinnitus (46 % of sample), and 85 % of the sample had at least one clinically significant hearing disorder, as verified by retrospective audiology reports (Tate et al. 2014).

Moreover, post-injury changes in hearing, such as hyperacusis (Capó-Aponte et al. 2012) can negatively impact engagement and participation in everyday activities for TBI patients. For example, Landon et al. (2012) described the phenomenological experiences of six TBI patients (mostly moderate-severe) who reported sensitivity to sounds, and found that a common theme was that patients avoided previously agreeable activities, such as going to a concert, church, or to a bar with friends, because of their aversion to loud sounds. Such findings offer support for a causal relationship between behavioral impairments (i.e., sensory-perceptual) and changes in participation in TBI.

Furthermore, these deficits may contribute to poor performance on cognitive tasks. For instance, Kim et al. (2012) used perfusion functional magnetic resonance imaging (fMRI) to examine cerebral blood flow during performance of attention and executive function tasks in moderate to severe TBI patients and healthy controls. TBI patients activated the occipital cortex bilaterally and left superior temporal cortex to a greater extent than controls, whereas these sensory areas were less perfused at rest compared to controls. As patients performed more poorly on the executive task than controls, the authors concluded that this pattern of activation may point to a contribution of sensory processing deficits to reduced cognitive task performance (Kim et al. 2012).

Thus, there is evidence for a range of impairments to sensory processing in moderate to severe TBI, and at least modest evidence that such changes influence cognitive processing. Directly relevant to the negative neuroplasticity framework are the findings that perceptual deficits such as hyperacusis and photosensitivity may lead to withdrawal from everyday activities and reduced participation. TBI-specific interventions to ameliorate sensory-perceptual deficits have yet to be reported. However, treatments utilized in non-TBI populations may have application (Anderson et al. 2013), and again, may serve to modify engagement and participation, with potential reductions in downstream negative learning and negative neuroplasticity.

### Weakened Neuromodulatory Control

Studies have examined the impact of TBI on dopamine, acetylcholine, serotonin, and norepinephrine. Patients with TBI (Bales et al. 2009) sustain changes in levels of dopamine, which have been linked with changes in certain cognitive abilities such as learning. Treatment with DA agonists (central nervous system stimulants such as methylphenidate as well as non-stimulant agonists) has been shown to improve attention, memory, and executive function in TBI patients (Writer and Schillerstrom 2009), and spatial memory performance in animal models of severe TBI (Rau et al. 2012). Treatment with the dopamine agonist amantadine also benefitted post-injury outcomes, specifically by increasing arousal in the acute phase of injury (Wheaton et al. 2009).

Acetylcholine dysregulation is also linked with cognitive impairment in TBI. Cholinergic nuclei in the brainstem and forebrain are susceptible to damage sustained from TBI (Arciniegas 2011a), and basal forebrain neurons are known to undergo degeneration as a result of TBI (Schliebs and Arendt 2011). Specifically, it is postulated that deficits in attention, memory, and executive functions such as working memory result from both acute stage increases and post-acute stage decreases in acetylcholine in the brain, based on studies that have assessed levels of acetylcholine with cerebrospinal fluid sampling as well as post-mortem analysis of TBI patients with severe injury (Arciniegas 2011b).

Decreased levels of serotonin have been observed after TBI (Writer and Schillerstrom 2009). However, few studies have found evidence for the efficacy of drugs that act on the serotonergic system to treat TBI-related cognitive deficits in humans (Banos et al. 2010), such as selective serotonin reuptake inhibitors, in contrast to findings in animal models of TBI, where research points to a positive effect of serotonin receptor agonist administration on spatial learning and memory after injury (Kline et al. 2012). There is also some evidence of the role of norepinephrine dysregulation in executive function (working memory) deficits in TBI (Kobori et al. 2011).

Taken together, these results show that weakened neuromodulatory control is observed after moderate to severe TBI. Importantly, some advances have been made in improving neuromodulatory function, which could ultimately serve to diminish some of the deleterious effects of compromised neuromodulation in chronic TBI; this, in turn, might help to offset negative neuroplastic change and neurodegenerative losses in this population.

### Negative Learning

As described above, TBI patients show reduced schedules of activity, deficits in sensory-perceptual processing that appear to interfere with cognitive functioning, and reduced neuromodulatory control. There is also some evidence for negative learning, that is, the bias towards simpler and more habitual cognitive processes at the expense of complex processing.

One relevant study examined performance of young adults with mild to severe TBI (a mean of 6 years post-injury) on a word recognition task in which participants were asked to judge whether words had been presented in a previously seen list, or were “new” words (Ozen et al. 2010). TBI patients were more susceptible to falsely recognizing familiar distractor words; these findings may be attributable to an increased reliance on the processing of familiar information as well as to preserved remote/autobiographical memory function in the context of impaired anterograde functions. Similarly, Arenth et al. (2012) administered a recall test (California Verbal Learning Test-II) to TBI patients and healthy controls and found that, in addition to displaying reduced recall of words compared to controls, TBI patients showed less evidence of using semantic clustering (considered to involve executive control functions) to retrieve words. As patients did not differ from controls in terms of use of serial clustering (considered an implicit strategy), the findings suggest a reliance on less effortful cognitive strategies in TBI (Arenth et al. 2012). These findings suggest that cognitive impairments may render various forms of more effortful processing either less effective or disproportionately difficult; either way, they are consequently avoided, and replaced where possible with simpler, less effortful mental processes.

As in the older adult literature, there is currently a paucity of direct evidence showing that negative learning leads to neuroplastic changes in the brain; to date, the finding that less self-reported EE is associated with greater hippocampal atrophy offers some preliminary support for this speculation (Miller et al. 2013).

## Potential Neural Mechanisms of Environmental Impoverishment, Negative Learning, and Neuroplastic Change

A number of neural mechanisms have been linked with neuroplastic changes that result from differential exposure to an enriched vs. impoverished environment. It logically follows that the same mechanisms that are involved in neuroplastic changes leading to desirable neural and cognitive outcomes under conditions of an enriched environment may be involved in negative neuroplastic changes associated with a dearth of such exposure. In this section, we outline a number of potential mechanisms that may underlie negative neuroplastic change.

Neuroplastic changes may arise through neurogenesis, particularly in the hippocampus. There is evidence in animals that thousands of neurons are generated daily in the adult hippocampus (see Shors et al. 2012 for a review). These “immature” neurons show greater mobility of spines (Toni et al. 2007) and are more likely to be integrated into neural circuits in the dentate gyrus of the hippocampus that underlies spatial memory, compared to mature neurons (Kee et al. 2007). Most relevant to the negative neuroplasticity framework, is the mounting evidence that degree of behavioural stimulation can either enhance or undermine the survivorship of newly generated neurons, as well as their integration into existing neuronal networks —a case of “use it or lose it” (Shors et al. 2012). Hippocampal volume loss in the chronic stages of moderate-severe TBI may be attributable to reduced proliferation or maintenance of newly generated neurons (in addition to losses of existing neuropil or cell-bodies in the hippocampus); thus, the findings of a relationship between behaviour and the survival of new hippocampal neurons—described below—provides support for a relationship between negative neuroplasticity and chronic stage hippocampal volume losses.

Accumulating studies indicate that new, complicated and challenging learning (as measured by number of trials to reach asymptote for example) results in greater maintenance of new hippocampal cells, whereas exposure to simple tasks in which learning does not occur does not result in enhanced maintenance (Curlik and Shors 2011; Dalla et al. 2007; Waddell and Shors 2008). For instance, survival of new neurons is enhanced in animals that take more trials to learn a trace conditioned eye-blink response, but do eventually learn the response (indicative of task challenge), as compared with animals who learn the task in a fewer number of trials (lesser challenge) or those animals who fail to learn the response (Curlik and Shors 2011; Dalla et al. 2007; Sisti et al. 2007). What is more, newly formed neurons that do not integrate into existing neural networks undergo apoptotic cell death, whereas neurons that make connections with surrounding networks through axonal sprouting are prevented from this fate by neurotrophic factors (Nicholson 2009). Thus, engagement in challenging cognitive tasks is one way in which death of newly formed neurons can be prevented. These findings suggest that in chronic TBI, negative learning – with reliance on more simple, routinized behaviours – may play a role in hippocampal volume losses. Here, negative learning may lead to reduced survival of new neurons, completing a vicious cycle in which ongoing new learning is increasingly hampered.

## Environmental Enrichment provides Opportunities for Intervention

EE confers a wide range of benefits to the brain. The cortex of rodents exposed to enriched environments during young adulthood show increased levels of neurotrophic factors and higher expression of genes controlling neuronal signalling/growth, RNA, and protein synthesis (Rampon et al. 2000a; Torasdotter et al. 1998, 1996). Also, enriched environments and new learning have been shown to increase neuropil in the cortex (Wallace et al. 2011), and result in structural changes in the dentate gyrus, cingulate cortex and corpus callosum (Blumenfeld-Katzir et al. 2011). These animal findings support the use of this apparently benign behavioural approach to enhance brain health/function in TBI.

More directly relevant to the current framework and the impact on neurodegeneration, EE has also been shown to offset the effects of a damaged hippocampus (for a review see Frick and Benoit 2010). For example, a study on mice with a deleted gene for *N*-methyl-D-aspartate receptor 1 subunit in the hippocampus CA1 region demonstrated that 3 h of enrichment a day for 2 months reduced the knock-out induced deficits. Specifically, the mice showed improved object recognition, improvements in learning a contextual fear response, and improved olfactory discrimination memory (Rampon et al. 2000b).

Such results, and many others, suggest that EE might have utility for breaking the downward spiral of negative neuroplastic change, by increasing participation and thereby preventing negative learning.

## Conclusions, Rehabilitation Implications, and Future Directions

The current paper argues that the Mahncke et al. (2006a, b) framework for aging related decline can be extrapolated to TBI, with the possibility that disuse-mediated negative neuroplastic change may underlie cognitive and neural declines associated in chronic stage TBI. Based on the negative neuroplasticity framework for older adults (Mahncke et al. 2006a, b), we outlined evidence for each of their factors in the moderate to severe TBI population. In this adapted provisional framework, we suggest that reduced schedules of activity and declines in sensory-perceptual and neuromodulatory function may combine to promote reliance on simpler cognitive processing leading to a reduced capacity to perform complex tasks at a high level in both of these populations (i.e., negative learning). We suggest that this downward spiral of negative neuroplastic change resulting in disuse of previously healthy neural structures may be a contributing factor to accelerated aging (Bigler 2013b), atrophy and white matter alterations that are observed in the chronic stages of moderate-severe TBI (Green et al. 2014; Adnan et al. 2013).

In extrapolating from the older adult framework to moderate-severe TBI, we note a rehabilitation-relevant caveat. Mahncke et al. (Mahncke et al. 2006a, b) postulate that the detrimental behavioural effects of negative neuroplasticity in part result from routinization of everyday activities. In TBI, as discussed by Frasca et al. (2013), there is evidence that establishment of daily routines actually increases the amount of social and cognitive activities in which TBI patients can engage. It may be that older adults spontaneously establish predictable routines that lack cognitive challenge, and so need to vary daily activities to provide challenge, whereas TBI patients require predictable routines, between which are stimulating and varied cognitive activities.

A strong implication of the concepts discussed in this paper is that neurorehabilitative efforts should be modified to (1) emphasize EE and, (2) continue into the chronic stage of injury. This stands in contrast to the standard of care, which emphasizes delivery of rehabilitation in the acute and sub-acute stages post-injury. The standard of care may be both theoretically and practically based. The findings of neurodegeneration are relatively new, and hence offsetting neurodegeneration is not yet a target of neurorehabilitation. Indeed, there may be a tacit assumption that early recovery gains are maintained into the chronic stages of injury given that many recovery studies—particularly those using group rather than frequency data—present recovery as asymptotic rather than quadratic, due in part to measurements within the first year of functioning only (Christensen et al. 2008). There is also an assumption of a therapeutic window, analogous to the critical periods, whereby therapy is considered to be most important during the period of spontaneous recovery (León-Carrión et al. 2013). These above issues, coupled with (i) limited financial resources of healthcare systems, and (ii) relatively greater clinical management issues during the acute and sub-acute stages of injury (e.g., wandering; aggression) that protected clinical environments can better handle than family caregivers, may also contribute to the emphasis on early stage rehabilitation at the expense of chronic stage needs.

Regarding the specific goals of therapies used in rehabilitation for TBI, the negative neuroplasticity framework reveals targets for intervention at multiple time points, and emphasizes the importance of engagement in enriched environments. An additional strength of the negative neuroplasticity framework is that it highlights the relationships between behavioural impairments, including altered sensory/perceptual processes, and environmental engagement. This again highlights new targets for intervention. For example, remediation of TBI-induced hearing loss might result in greater fidelity of auditory representations, resulting in greater engagement in day-to-day life. The framework also speaks to the need for a multi-faceted approach to treating TBI, and in particular the need for approaches that avert or remediate negative neuroplastic changes/neurodegeneration. For example, interventions might emphasize treatment of the primary impairments of TBI (e.g., cognitive, sensory-perceptual, mood and physical functioning), but should utilize creative approaches to incorporate the active ingredients of EE, including intensive and continuously novel, challenging and engaging activities. This would ideally entail attunement of rehabilitation programs/therapists to approaches to offsetting the moderators of reduced participation discussed (e.g., lack of transportation to stimulating environments). The development of more tele-therapy approaches, especially group-based ones that confer social stimulation, might be helpful in this regard.

We note that there are important gaps in empirical support for aspects of this preliminary framework. For instance, the idea that an increased reliance on familiar and routine behaviours leads to attenuated activity in brain networks that were previously engaged in complex cognitive activities has not been directly tested in TBI patients. As well, there are factors that may have a direct influence on brain structure and function in the chronic stages of injury, such as depression, anxiety, stress and neuroinflammation, which are not moderated through negative learning or negative neuroplasticity. As these are particularly relevant to TBI patients (Dikmen et al. 2004; Hart et al. 2012; Gould et al. 2011; Arundine et al. 2012) it may be prudent to address, for example, psychological disorders that are associated with reduced activity levels (i.e., depression) in tandem with encouraging increased cognitive stimulation.

## How the Negative Neuroplasticity Framework Can Guide Future Research Efforts Into Neurodegeneration and Treatment in TBI

As described above, there are empirical gaps in this preliminary framework. Future longitudinal research is needed to investigate the degree to which increased reliance on simpler modes of cognitive processing (e.g., less effortful strategies, disproportionate use of familiar or existing autobiographical knowledge) is associated with degree of subsequent cognitive or neural decline. For instance, relations between strategy use (simple vs. complex) in memory tasks and changes in brain volume or white matter integrity could be examined.

Concerning the causal relation between negative neuroplastic change and neural atrophy in chronic TBI that is proposed in the negative neuroplasticity framework, a follow-up study to Miller et al. (2013) would be of value. Here, we found that hippocampal atrophy was negatively associated with self-reported EE. A prospective, longitudinal study, in which levels and type of EE are manipulated (e.g., cognitive, social and physical; EE targeted to specific cognitive impairments vs. more “generic” EE programs such as computerized brain games), would help to identify the causal contributions of EE. Here, pre-post quantitative magnetic resonance imaging (MRI) of the hippocampi would be elucidating; visualization of the dentate gyrus (on 7 T structural MRI, for example) would help to discriminate between the respective contributions of actual cell death, losses to the neuropil (Kerchner et al. 2013), and reduced neurogenesis as contributors to activity-dependent hippocampal volume losses in chronic TBI. Functional imaging measures (e.g., longitudinal functional connectivity changes) may provide earlier and more sensitive responses to EE manipulations, as would a diffusion tensor imaging examination of white matter integrity.

A fine-grained time-course of study could allow for temporal relationships between changes in white vs. grey matter to be revealed, and their relationship to EE manipulations. For any study, a randomized control design with an activity control group is optimal, along with control for depression and stress given the known impact of both on the hippocampi (Fotuhi et al. 2012).

As previous studies have largely relied on self-reported activity levels, it would be of high value to examine the effects of interventions that specifically provide continuously novel, complex and intensive cognitive activities on brain changes, in comparison with active control conditions, such as used in studies of older adults (Park et al. 2014). In the perceptual domain, pre-post effects of auditory training programs (e.g., Anderson et al. 2013) in TBI on changes in auditory cortex function/volume and in hearing ability could be examined.

Future studies could also seek to clarify the neural basis of training-related improvements in cognitive function in TBI patients to determine whether cognitive training/cognitive rehabilitation programs can encourage neurogenesis and re-activate neurons/networks vulnerable to atrophy through disuse. Given that neurogenesis in the hippocampus may be spurred by challenging learning tasks (Shors et al. 2012), a protocol that is continually novel and challenging may also spur neurogenesis in chronic stage TBI patients. This type of protocol may also help to maintain neurons spared by injury, alleviating effects of negative neuroplasticity that may have occurred. For instance, new neurons that survive because of cognitive training may become integrated into existing neural networks, thereby supporting and improving neuronal function, which could support further development of cognitive abilities. Such alterations may manifest as changes in task-related functional neural networks—as assessed by fMRI—that mimic more closely those of healthy individuals. They may also manifest as growth or relative preservation of brain areas associated with the specific tasks being trained (e.g., preservation of hippocampal volume as a result of memory training programs).

## Conclusions

We conclude that the Mahncke et al. (2006a, b) framework developed for older adults has value for the chronic TBI population. The adaptation of the older adult framework that we present offers testable hypotheses, and ideas for novel points of intervention for clinicians that may help to offset a downward neurological course of chronic moderate-severe TBI. Given the prevalence of patients suffering in the chronic stages of injury, and the relatively limited attention paid to this population, this adapted preliminary framework may prove useful by encouraging ongoing research that is specific to chronic TBI.

## References

[CR1] Adnan A, Crawley A, Mikulis D, Moscovitch M, Colella B, Green R (2013). Moderate-severe traumatic brain injury causes delayed loss of white matter integrity: Evidence of fornix deterioration in the chronic stage of injury. Brain Injury.

[CR2] Andelic N, Sigurdardottir S, Schanke AK, Sandvik L, Sveen U, Roe C (2010). Disability, physical health and mental health 1 year after traumatic brain injury. Disability and Rehabilitation.

[CR3] Anderson TM, Knight RG (2010). The long-term effects of traumatic brain injury on the coordinative function of the central executive. Journal of Clinical and Experimental Neuropsychology.

[CR4] Anderson S, White-Schwoch T, Parbery-Clark A, Kraus N (2013). Reversal of age-related neural timing delays with training. Proceedings of the National Academy of Sciences of the United States of America.

[CR5] Andrew MK, Rockwood K (2010). Social vulnerability predicts cognitive decline in a prospective cohort of older Canadians. Alzheimer’s & Dementia.

[CR6] Angel L, Bastin C, Genon S, Balteau E, Phillips C, Luxen A (2013). Differential effects of aging on the neural correlates of recollection and familiarity. Cortex.

[CR7] Arciniegas, D. B. (2011a). Cholinergic dysfunction and cognitive impairment after traumatic brain injury. Part 1: the structure and function of cerebral cholinergic systems. *Journal of Head Trauma Rehabilitation, 26*(1), 98–101.. 10.1097/HTR.0b013e31820516cb 21209567

[CR8] Arciniegas, D. B. (2011b). Cholinergic dysfunction and cognitive impairment after traumatic brain injury. Part 2: evidence from basic and clinical investigations. *Journal of Head Trauma Rehabilitation, 26*(4), 319–323. 10.1097/HTR.0b013e31821ebfb3 21734513

[CR9] Arenth PM, Russell KC, Scanlon JM, Kessler LJ, Ricker JH (2012). Encoding and recognition after traumatic brain injury: neuropsychological and functional magnetic resonance imaging findings. Journal of Clinical and Experimental Neuropsychology.

[CR10] Arenth PM, Russell KC, Scanlon JM, Kessler LJ, Ricker JH (2013). Corpus callosum integrity and neuropsychological performance after traumatic brain injury: a diffusion tensor imaging study. Journal of Head Trauma Rehabilitation.

[CR11] Arundine A, Bradbury CL, Dupuis K, Dawson DR, Ruttan LA, Green REA (2012). Cognitive behavior therapy after acquired brain injury: Maintenance of therapeutic benefits at 6 months posttreatment. Journal of Head Trauma Rehabilitation.

[CR12] Bäckman L, Lindenberger U, Li SC, Nyberg L (2010). Linking cognitive aging to alterations in dopamine neurotransmitter functioning: recent data and future avenues. Neuroscience and Biobehavioral Reviews.

[CR13] Bäckman L, Karlsson S, Fischer H, Karlsson P, Brehmer Y, Rieckmann A (2011). Dopamine D(1) receptors and age differences in brain activation during working memory. Neurobiology of Aging.

[CR14] Bales JW, Wagner AK, Kline AE, Dixon CE (2009). Persistent cognitive dysfunction after traumatic brain injury: a dopamine hypothesis. Neuroscience and Biobehavioral Reviews.

[CR15] Banos JH, Novack TA, Brunner R, Renfroe S, Lin HY, Meythaler J (2010). Impact of early administration of sertraline on cognitive and behavioral recovery in the first year after moderate to severe traumatic brain injury. Journal of Head Trauma Rehabilitation.

[CR16] Bashore TR, Ridderinkhof KR (2002). Older age, traumatic brain injury, and cognitive slowing: some convergent and divergent findings. Psychological Bulletin.

[CR17] Bernstein, L. (1972). The reversibility of learning deficits in early environmentally restricted rats as a function of amount of experience in later life. *Journal Psychosomatics Research, 16*, 71–73. 10.1016/0022-3999(72)90027-x 5062049

[CR18] Bier N, Dutil E, Couture M (2009). Factors affecting leisure participation after a traumatic brain injury: an exploratory study. Journal of Head Trauma Rehabilitation.

[CR19] Bigler ED (2013). Neuroinflammation and the dynamic lesion in traumatic brain injury. Brain.

[CR20] Bigler ED (2013). Traumatic brain injury, neuroimaging, and neurodegeneration. Frontiers in Human Neuroscience.

[CR21] Bigler ED, Maxwell WL (2011). Neuroimaging and neuropathology of TBI. NeuroRehabilitation.

[CR22] Bigler ED, Blatter DD, Anderson CV, Johnson SC, Gale SD, Hopkins RO (1997). Hippocampal volume in normal aging and traumatic brain injury. AJNR. American Journal of Neuroradiology.

[CR23] Blatter DD, Bigler ED, Gale SD, Johnson SC, Anderson CV, Burnett BM (1997). MR-based brain and cerebrospinal fluid measurement after traumatic brain injury: correlation with neuropsychological outcome. AJNR. American Journal of Neuroradiology.

[CR24] Blumenfeld-Katzir T, Pasternak O, Dagan M, Assaf Y (2011). Diffusion MRI of structural brain plasticity induced by a learning and memory task. PloS One.

[CR25] Bohbot VD, McKenzie S, Konishi K, Fouquet C, Kurdi V, Schachar R (2012). Virtual navigation strategies from childhood to senescence: evidence for changes across the life span. Frontiers in Aging Neuroscience.

[CR26] Bondi CO, Klitsch KC, Leary JB, Kline AE (2014). Environmental enrichment as a viable neurorehabilitation strategy for experimental traumatic brain injury. Journal of Neurotrauma.

[CR27] Bouazzaoui B, Isingrini M, Fay S, Angel L, Vanneste S, Clarys D (2010). Aging and self-reported internal and external memory strategy uses: the role of executive functioning. Acta Psychologica.

[CR28] Bouisson J (2002). Routinization preferences, anxiety, and depression in an elderly French sample. Journal of Aging Studies.

[CR29] Bouisson J, Swendsen J (2003). Routinization and emotional well-being: an experience sampling investigation in an elderly French sample. Journals of Gerontology. Series B, Psychological Sciences and Social Sciences.

[CR30] Buchman AS, Boyle PA, Wilson RS, Fleischman DA, Leurgans S, Bennett DA (2009). Association between late-life social activity and motor decline in older adults. Archives of Internal Medicine.

[CR31] Bulinski L (2010). Social reintegration of TBI patients: a solution to provide long-term support. Medical Science Monitor.

[CR32] Burzynska AZ, Preuschhof C, Backman L, Nyberg L, Li SC, Lindenberger U (2010). Age-related differences in white matter microstructure: region-specific patterns of diffusivity. NeuroImage.

[CR33] Cabeza R (2002). Hemispheric asymmetry reduction in older adults: the HAROLD model. Psychology and Aging.

[CR34] Campbell KL, Grady CL, Ng C, Hasher L (2012). Age differences in the frontoparietal cognitive control network: implications for distractibility. Neuropsychologia.

[CR35] Campbell, K. L., Grigg, O., Saverino, C., Churchill, N., & Grady, C. L. (2013). Age differences in the intrinsic functional connectivity of default network subsystems. *Frontiers in Aging Neuroscience, 5*. doi:10.3389/fnagi.2013.00073. 10.3389/fnagi.2013.00073 PMC3827623 24294203

[CR36] Capó-Aponte JE, Urosevich TG, Temme LA, Tarbett AK, Sanghera NK (2012). Visual dysfunctions and symptoms during the subacute stage of blast-induced mild traumatic brain injury. Military Medicine.

[CR37] Cashdollar N, Fukuda K, Bocklage A, Aurtenetxe S, Vogel EK, Gazzaley A (2013). Prolonged disengagement from attentional capture in normal aging. Psychology and Aging.

[CR38] Cazalis F, Feydy A, Valabregue R, Pelegrini-Issac M, Pierot L, Azouvi P (2006). fMRI study of problem-solving after severe traumatic brain injury. Brain Injury.

[CR39] Chowdhury R, Guitart-Masip M, Lambert C, Dayan P, Huys Q, Duzel E (2013). Dopamine restores reward prediction errors in old age. Nature Neuroscience.

[CR40] Chowdhury R, Guitart-Masip M, Lambert C, Dolan RJ, Duzel E (2013). Structural integrity of the substantia nigra and subthalamic nucleus predicts flexibility of instrumental learning in older-age individuals. Neurobiology of Aging.

[CR41] Christensen BK, Colella B, Inness E, Hebert D, Monette G, Bayley M (2008). Recovery of cognitive function after traumatic brain injury: a multilevel modeling analysis of Canadian outcomes. Archives of Physical Medicine and Rehabilitation.

[CR42] Clay OJ, Edwards JD, Ross LA, Okonkwo O, Wadley VG, Roth DL (2009). Visual function and cognitive speed of processing mediate age-related decline in memory span and fluid intelligence. Journal of Aging and Health.

[CR43] Cliff M, Joyce DW, Lamar M, Dannhauser T, Tracy DK, Shergill SS (2013). Aging effects on functional auditory and visual processing using fMRI with variable sensory loading. Cortex.

[CR44] Clinard CG, Tremblay KL, Krishnan AR (2010). Aging alters the perception and physiological representation of frequency: evidence from human frequency-following response recordings. Hearing Research.

[CR45] Cornwell B (2011). Age trends in daily social contact patterns. Research on Aging.

[CR46] Craik FI, Rose NS (2012). Memory encoding and aging: a neurocognitive perspective. Neuroscience and Biobehavioral Reviews.

[CR47] Curlik DM, Shors TJ (2011). Learning increases the survival of newborn neurons provided that learning is difficult to achieve and successful. Journal of Cognitive Neuroscience.

[CR48] Dalla C, Bangasser DA, Edgecomb C, Shors TJ (2007). Neurogenesis and learning: acquisition and asymptotic performance predict how many new cells survive in the hippocampus. Neurobiology of Learning and Memory.

[CR49] Der-Avakian A, Markou A (2012). The neurobiology of anhedonia and other reward-related deficits. Trends in Neurosciences.

[CR50] Dikmen SS, Bombardier CH, MacHamer JE, Fann JR, Temkin NR (2004). Natural history of depression in traumatic brain injury. Archives of Physical Medicine and Rehabilitation.

[CR51] Dorey, C. K., & Blanks, J. C. (2009). *Sensory aging: vision* (Handbook of the neuroscience of aging): Burlington: Elsevier/Academic Press.

[CR52] Dosher B, Lu Z-L (2009). Hebbian reweighting on stable representations in perceptual learning. Learning & Perception.

[CR53] Dumitriu D, Hao J, Hara Y, Kaufmann J, Janssen WG, Lou W (2010). Selective changes in thin spine density and morphology in monkey prefrontal cortex correlate with aging-related cognitive impairment. Journal of Neuroscience.

[CR54] Düzel S, Münte TF, Lindenberger U, Bunzeck N, Schutze H, Heinze HJ (2010). Basal forebrain integrity and cognitive memory profile in healthy aging. Brain Research.

[CR55] Edmonds EC, Glisky EL, Bartlett JC, Rapcsak SZ (2012). Cognitive mechanisms of false facial recognition in older adults. Psychology and Aging.

[CR56] Edwards JD, Lunsman M, Perkins M, Rebok GW, Roth DL (2009). Driving cessation and health trajectories in older adults. Journals of Gerontology Series A: Biological Sciences and Medical Sciences.

[CR57] Erickson KI, Prakash RS, Voss MW, Chaddock L, Heo S, McLaren M (2010). Brain-derived neurotrophic factor is associated with age-related decline in hippocampal volume. Journal of Neuroscience.

[CR58] Eskes GA, Longman S, Brown AD, McMorris CA, Langdon KD, Hogan DB (2010). Contribution of physical fitness, cerebrovascular reserve and cognitive stimulation to cognitive function in post-menopausal women. Frontiers in Aging Neuroscience.

[CR59] Evans JJ, Bateman A, Turner G, Green R (2008). Research digest. Neuropsychological Rehabilitation.

[CR60] Farbota KD, Bendlin BB, Alexander AL, Rowley HA, Dempsey RJ, Johnson SC (2012). Longitudinal diffusion tensor imaging and neuropsychological correlates in traumatic brain injury patients. Frontiers in Human Neuroscience.

[CR61] Farbota KD, Sodhi A, Bendlin BB, McLaren DG, Xu G, Rowley HA (2012). Longitudinal volumetric changes following traumatic brain injury: a tensor-based morphometry study. Journal of the International Neuropsychological Society.

[CR62] Fleming J, Nalder E, Alves-Stein S, Cornwell P (2013). The effect of environmental barriers on community integration for individuals with moderate to severe traumatic brain injury. Journal of Head Trauma Rehabilitation.

[CR63] Frasca D, Tomaszczyk J, McFadyen BJ, Green RE (2013). Traumatic brain injury and post-acute decline: what role does environmental enrichment play? A scoping review. Frontiers in Human Neuroscience.

[CR64] Frick KM, Benoit JD (2010). Use it or lose it: environmental enrichment as a means to promote successful cognitive aging. ScientificWorldJournal.

[CR65] Fung HH, Carstensen LL (2006). Goals change when life’s fragility is primed: lessons learned from older adults, the September 11 attacks and Sars. Social Cognition.

[CR66] Fotuhi, M., Do, D., & Jack, C. (2012). Modifiable factors that alter the size of the hippocampus with ageing. *Nature Reviews Neurology, 8*(4), 189–202. 10.1038/nrneurol.2012.27 22410582

[CR67] Gao X, Deng P, Xu ZC, Chen J (2011). Moderate traumatic brain injury causes acute dendritic and synaptic degeneration in the hippocampal dentate gyrus. PloS One.

[CR68] Gold BT, Kim C, Johnson NF, Kryscio RJ, Smith CD (2013). Lifelong bilingualism maintains neural efficiency for cognitive control in aging. Journal of Neuroscience.

[CR69] Gonoi W, Abe O, Yamasue H, Yamada H, Masutani Y, Takao H (2010). Age-related changes in regional brain volume evaluated by atlas-based method. Neuroradiology.

[CR70] Goodrich GL, Flyg HM, Kirby JE, Chang CY, Martinsen GL (2013). Mechanisms of TBI and visual consequences in military and veteran populations. Optometry and Vision Science.

[CR71] Gould KR, Ponsford JL, Johnston L, Schönberger M (2011). The nature, frequency and course of psychiatric disorders in the first year after traumatic brain injury: a prospective study. Psychological Medicine.

[CR72] Green RE, Colella B, Hebert DA, Bayley M, Kang HS, Till C (2008). Prediction of return to productivity after severe traumatic brain injury: investigations of optimal neuropsychological tests and timing of assessment. Archives of Physical Medicine and Rehabilitation.

[CR73] Green, R. E. A., Colella, B., Maller, J. J., Bayley, M., Glazer, J., & Mikulis, D. R. (2014). Scale and pattern of atrophy in the chronic stages of moderate-severe TBI. [Original Research]. *Frontiers in Human Neuroscience, 8*. doi:10.3389/fnhum.2014.00067. 10.3389/fnhum.2014.00067 PMC3978360 24744712

[CR74] Greenberg G, Mikulis DJ, Ng K, DeSouza D, Green RE (2008). Use of diffusion tensor imaging to examine subacute white matter injury progression in moderate to severe traumatic brain injury. Archives of Physical Medicine and Rehabilitation.

[CR75] Greenwald BD, Kapoor N, Singh AD (2012). Visual impairments in the first year after traumatic brain injury. Brain Injury.

[CR76] Hamm, R., Temple, M., O'dell, D., Pike, B. & Lyeth, B. (1996). Exposure to environmental complexity promotes recovery of cognitive function after traumtic brain injury. *Journal of Neurotrauma, 13*, 41–47. 10.1089/neu.1996.13.41 8714862

[CR77] Hammond FM, Grattan KD, Sasser H, Corrigan JD, Rosenthal M, Bushnik T (2004). Five years after traumatic brain injury: a study of individual outcomes and predictors of change in function. NeuroRehabilitation.

[CR78] Hannan AJ (2014). Review: environmental enrichment and brain repair: harnessing the therapeutic effects of cognitive stimulation and physical activity to enhance experience-dependent plasticity. Neuropathology and Applied Neurobiology.

[CR79] Hart, T., Hoffman, J. M., Pretz, C., Kennedy, R., Clark, A. N., Brenner, L. A. (2012). A longitudinal study of major and minor depression following traumatic brain injury. *Archives of Physical Medicine and Rehabilitation, 93*, 1343–9. 10.1016/j.apmr.2012.03.036 22840833

[CR80] Hawthorne G, Gruen RL, Kaye AH (2009). Traumatic brain injury and long-term quality of life: findings from an Australian study. Journal of Neurotrauma.

[CR81] Hebb DO (1947). The effects of early experience on problem-solving at maturity. American Psychologist.

[CR82] Hebb, D. O. (1949). *The organization of behavior*. New York: Wiley.

[CR83] Hedman AM, van Haren NE, Schnack HG, Kahn RS, Hulshoff Pol HE (2012). Human brain changes across the life span: a review of 56 longitudinal magnetic resonance imaging studies. Human Brain Mapping.

[CR84] Himanen L, Portin R, Isoniemi H, Helenius H, Kurki T, Tenovuo O (2006). Longitudinal cognitive changes in traumatic brain injury: a 30-year follow-up study. Neurology.

[CR85] Humes LE, Busey TA, Craig J, Kewley-Port D (2013). Are age-related changes in cognitive function driven by age-related changes in sensory processing?. Attention, Perception, & Psychophysics.

[CR86] Hyder AA, Wunderlich CA, Puvanachandra P, Gururaj G, Kobusingye OC (2007). The impact of traumatic brain injuries: a global perspective. NeuroRehabilitation.

[CR87] Jacoby LL, Rhodes MG (2006). False remembering in the aged. Current Directions in Psychological Science.

[CR88] James BD, Wilson RS, Barnes LL, Bennett DA (2011). Late-life social activity and cognitive decline in old age. Journal of the International Neuropsychological Society.

[CR89] Johansson, B. B., Ohlsson, A. L. (1996). Environment, social interaction, and physical activity as determinants of functional outcome after cerebral infarction in the rat. *Experimental Neurology 139*, 322–327. 10.1006/exnr.1996.0106 8654535

[CR90] Johnson VE, Stewart JE, Begbie FD, Trojanowski JQ, Smith DH, Stewart W (2013). Inflammation and white matter degeneration persist for years after a single traumatic brain injury. Brain.

[CR91] Kasahara M, Menon DK, Salmond CH, Outtrim JG, Tavares JV, Carpenter TA (2011). Traumatic brain injury alters the functional brain network mediating working memory. Brain Injury.

[CR92] Kavé G, Mashal N (2012). Age-related differences in word-retrieval but not in meaning generation. Neuropsychology, Development, and Cognition. Section B, Aging, Neuropsychology and Cognition.

[CR93] Kee N, Teixeira CM, Wang AH, Frankland PW (2007). Preferential incorporation of adult-generated granule cells into spatial memory networks in the dentate gyrus. Nature Neuroscience.

[CR94] Kerchner GA, Bernstein JD, Fenesy MC, Deutsch GK, Saranathan M, Zeineh MM (2013). Shared vulnerability of two synaptically-connected medial temporal lobe areas to age and cognitive decline: a seven tesla magnetic resonance imaging study. Journal of Neuroscience.

[CR95] Kersten AW, Earles JL (2010). Effects of aging, distraction, and response pressure on the binding of actors and actions. Psychology and Aging.

[CR96] Kim J, Whyte J, Patel S, Europa E, Slattery J, Coslett HB (2012). A perfusion fMRI study of the neural correlates of sustained-attention and working-memory deficits in chronic traumatic brain injury. Neurorehabilitation and Neural Repair.

[CR97] Kirchhoff BA, Gordon BA, Head D (2014). Prefrontal gray matter volume mediates age effects on memory strategies. NeuroImage.

[CR98] Kline AE, Olsen AS, Sozda CN, Hoffman AN, Cheng JP (2012). Evaluation of a combined treatment paradigm consisting of environmental enrichment and the 5-HT1A receptor agonist buspirone after experimental traumatic brain injury. Journal of Neurotrauma.

[CR99] Klinkenberg I, Sambeth A, Blokland A (2011). Acetylcholine and attention. Behavioural Brain Research.

[CR100] Kobayashi S, Ohashi Y, Ando S (2002). Effects of enriched environments with different durations and starting times on learning capacity during aging in rats assessed by a refined procedure of the Hebb-Williams maze task. Journal of Neuroscience Research.

[CR101] Kobori N, Hu B, Dash PK (2011). Altered adrenergic receptor signaling following traumatic brain injury contributes to working memory dysfunction. Neuroscience.

[CR102] Koshimori Y, Johns K, Green REA (2009). A guide for hearing healthcare providers to characteristics of traumatic brain injury. Hearing Journal.

[CR103] Köstering L, Stahl C, Leonhart R, Weiller C, Kaller CP (2013). Development of planning abilities in normal aging: differential effects of specific cognitive demands. Developmental Psychology.

[CR104] Krebs RM, Heipertz D, Schuetze H, Duzel E (2011). Novelty increases the mesolimbic functional connectivity of the substantia nigra/ventral tegmental area (SN/VTA) during reward anticipation: evidence from high-resolution fMRI. NeuroImage.

[CR105] Kumar A, Loane DJ (2012). Neuroinflammation after traumatic brain injury: opportunities for therapeutic intervention. Brain, Behavior, and Immunity.

[CR106] Laguë-Beauvais M, Brunet J, Gagnon L, Lesage F, Bherer L (2013). A fNIRS investigation of switching and inhibition during the modified Stroop task in younger and older adults. NeuroImage.

[CR107] Landon J, Shepherd D, Stuart S, Theadom A, Freundlich S (2012). Hearing every footstep: noise sensitivity in individuals following traumatic brain injury. Neuropsychological Rehabilitation.

[CR108] León-Carrión J, MacHuca-Murga F, Solís-Marcos I, León-Domínguez U, Domínguez-Morales MDR (2013). The sooner patients begin neurorehabilitation, the better their functional outcome. Brain Injury.

[CR109] Leunissen I, Coxon JP, Geurts M, Caeyenberghs K, Michiels K, Sunaert S (2013). Disturbed cortico-subcortical interactions during motor task switching in traumatic brain injury. Human Brain Mapping.

[CR110] Lew, H. L., Jerger J. F., Guillory, S. B., Henry, J. A. (2007). Auditory dysfunction in traumatic brain injury. *The Journal of Rehabilitation Research and Development, 44*(7), 921–928. doi:10.1682/jrrd.2007.09.0140. 10.1682/jrrd.2007.09.0140 18075949

[CR111] Lew HL, Weihing J, Myers PJ, Pogoda TK, Goodrich GL (2010). Dual sensory impairment (DSI) in traumatic brain injury (TBI)–An emerging interdisciplinary challenge. NeuroRehabilitation.

[CR112] Lin FR, Ferrucci L, Metter EJ, An Y, Zonderman AB, Resnick SM (2011). Hearing loss and cognition in the Baltimore Longitudinal Study of Aging. Neuropsychology.

[CR113] Lu, L., Bao, G., Chen, H., Xia, P., Fan, X., Zhang, J. et al. (2003). Modification of hippocampal neurogenesis and neuroplasticity by social environments. *Experimental Neurology, 183*(2), 600–609. doi:10.1016/s0014-4886(03)00248-6. 10.1016/s0014-4886(03)00248-6 14552901

[CR114] Luo L, Craik FI, Moreno S, Bialystok E (2013). Bilingualism interacts with domain in a working memory task: evidence from aging. Psychology and Aging.

[CR115] Mahncke HW, Bronstone A, Merzenich MM (2006). Brain plasticity and functional losses in the aged: scientific bases for a novel intervention. Progress in Brain Research.

[CR116] Mahncke HW, Connor BB, Appelman J, Ahsanuddin ON, Hardy JL, Wood RA (2006). Memory enhancement in healthy older adults using a brain plasticity-based training program: a randomized, controlled study. Proceedings of the National Academy of Sciences of the United States of America.

[CR117] Mangels JA, Craik FI, Levine B, Schwartz ML, Stuss DT (2002). Effects of divided attention on episodic memory in chronic traumatic brain injury: a function of severity and strategy. Neuropsychologia.

[CR118] Masel BE, DeWitt DS (2010). Traumatic brain injury: a disease process, not an event. Journal of Neurotrauma.

[CR119] Mata R, von Helversen B, Rieskamp J (2010). Learning to choose: cognitive aging and strategy selection learning in decision making. Psychology and Aging.

[CR120] McDaniel, M. A., Einstein, G. O., & Jacoby, L. L. (2008). New considerations in aging and memory: The glass may be half full. In F. I. M. Craik & T. A. Salthouse (Eds.), *The handbook of aging and cognition, 3rd edition* (pp. 251–310). New York: Psychology Press.

[CR121] McGarry LJ, Thompson D, Millham FH, Cowell L, Snyder PJ, Lenderking WR (2002). Outcomes and costs of acute treatment of traumatic brain injury. Journal of Trauma.

[CR122] McGinnis D (2012). Susceptibility to distraction during reading in young, young-old, and old-old adults. Experimental Aging Research.

[CR123] Mezuk B, Rebok GW (2008). Social integration and social support among older adults following driving cessation. Journals of Gerontology - Series B Psychological Sciences and Social Sciences.

[CR124] Miller LS, Colella B, Mikulis D, Maller J, Green RE (2013). Environmental enrichment may protect against hippocampal atrophy in the chronic stages of traumatic brain injury. Frontiers in Human Neuroscience.

[CR125] Millis SR, Rosenthal M, Novack TA, Sherer M, Nick TG, Kreutzer JS (2001). Long-term neuropsychological outcome after traumatic brain injury. Journal of Head Trauma Rehabilitation.

[CR126] Morrison JH, Baxter MG (2012). The ageing cortical synapse: hallmarks and implications for cognitive decline. Nature Reviews Neuroscience.

[CR127] Morton MV, Wehman P (1995). Psychosocial and emotional sequelae of individuals with traumatic brain injury: a literature review and recommendations. Brain Injury.

[CR128] Munjal SK, Panda NK, Pathak A (2010). Relationship between severity of traumatic brain injury (TBI) and extent of auditory dysfunction. Brain Injury.

[CR129] National Institutes of Health (1998). Rehabilitation of persons with traumatic brain injury. National Institutes of Health (NIH) Consensus Statement.

[CR130] Ng K, Mikulis DJ, Glazer J, Kabani N, Till C, Greenberg G (2008). Magnetic resonance imaging evidence of progression of subacute brain atrophy in moderate to severe traumatic brain injury. Archives of Physical Medicine and Rehabilitation.

[CR131] Nicholson DW (2009). Neuroscience: good and bad cell death. Nature.

[CR132] Nimrod G, Janke MC, Kleiber DA (2009). Expanding, reducing, concentrating and diffusing: activity patterns of recent retirees in the United States. Leisure Sciences.

[CR133] Novak DL, Mather M (2007). Aging and variety seeking. Psychology and Aging.

[CR134] Ord JS, Greve KW, Bianchini KJ, Aguerrevere LE (2010). Executive dysfunction in traumatic brain injury: the effects of injury severity and effort on the Wisconsin Card Sorting Test. Journal of Clinical and Experimental Neuropsychology.

[CR135] Ostreicher ML, Moses SN, Rosenbaum RS, Ryan JD (2010). Prior experience supports new learning of relations in aging. Journals of Gerontology. Series B, Psychological Sciences and Social Sciences.

[CR136] Ozen LJ, Skinner EI, Fernandes MA (2010). Rejecting familiar distracters during recognition in young adults with traumatic brain injury and in healthy older adults. Journal of the International Neuropsychological Society.

[CR137] Palacios EM, Sala-Llonch R, Junque C, Fernandez-Espejo D, Roig T, Tormos JM (2013). Long-term declarative memory deficits in diffuse TBI: correlations with cortical thickness, white matter integrity and hippocampal volume. Cortex.

[CR138] Pannese E (2011). Morphological changes in nerve cells during normal aging. Brain Structure and Function.

[CR139] Park DC, Lodi-Smith J, Drew L, Haber S, Hebrank A, Bischof GN (2014). The impact of sustained engagement on cognitive function in older adults: the synapse project. Psychological Science.

[CR140] Peelle, J. E., Troiani, V., Grossman, M., Wingfield, A. (2011). Hearing loss in older adults affects neural systems supporting speech comprehension. *Journal of Neuroscience, 31*(35), 12638–12643. 10.1523/JNEUROSCI.2559-11.2011 PMC3175595 21880924

[CR141] Peters A, Kemper T (2012). A review of the structural alterations in the cerebral hemispheres of the aging rhesus monkey. Neurobiology of Aging.

[CR142] Petrosini L, De Bartolo P, Foti F, Gelfo F, Cutuli D, Leggio MG (2009). On whether the environmental enrichment may provide cognitive and brain reserves. Brain Research Reviews.

[CR143] Polinder S, Meerding WJ, van Baar ME, Toet H, Mulder S, van Beeck EF (2005). Cost estimation of injury-related hospital admissions in 10 European countries. The Journal of Trauma: Injury, Infection, and Critical Care.

[CR144] Povlishock JT, Katz DI (2005). Update of neuropathology and neurological recovery after traumatic brain injury. Journal of Head Trauma Rehabilitation.

[CR145] Pushkar D, Chaikelson J, Conway M, Etezadi J, Giannopoulus C, Li K (2010). Testing continuity and activity variables as predictors of positive and negative affect in retirement. Journals of Gerontology. Series B, Psychological Sciences and Social Sciences.

[CR146] Ramlackhansingh AF, Brooks DJ, Greenwood RJ, Bose SK, Turkheimer FE, Kinnunen KM (2011). Inflammation after trauma: Microglial activation and traumatic brain injury. Annals of Neurology.

[CR147] Rampon C, Jiang CH, Dong H, Tang YP, Lockhart DJ, Schultz PG (2000). Effects of environmental enrichment on gene expression in the brain. Proceedings of the National Academy of Sciences of the United States of America.

[CR148] Rampon C, Tang YP, Goodhouse J, Shimizu E, Kyin M, Tsien JZ (2000). Enrichment induces structural changes and recovery from nonspatial memory deficits in CA1 NMDAR1-knockout mice. Nature Neuroscience.

[CR149] Rau TF, Kothiwal AS, Rova AR, Brooks DM, Poulsen DJ (2012). Treatment with low-dose methamphetamine improves behavioral and cognitive function after severe traumatic brain injury. Journal of Trauma and Acute Care Surgery.

[CR150] Raz N, Ghisletta P, Rodrigue KM, Kennedy KM, Lindenberger U (2010). Trajectories of brain aging in middle-aged and older adults: regional and individual differences. NeuroImage.

[CR151] Resch JA, Villarreal V, Johnson CL, Elliott TR, Kwok O-M, Berry JW (2009). Trajectories of life satisfaction in the first 5 years following traumatic brain injury. Rehabilitation Psychology.

[CR152] Rodgers MK, Sindone JA, Moffat SD (2012). Effects of age on navigation strategy. Neurobiology of Aging.

[CR153] Rosenzweig, M. (1966). Environmental complexity, cerebral change, and behavior. *American Psychologist, 21*, 321–332. 10.1037/h0023555 5910063

[CR154] Rosenzweig, M. and Bennett, E. (1996). Psychobiology of plasticity: effects of training and experience on brain and behaviour. *Behavioural Brain Research, 78*, 57–65. 10.1016/0166-4328(95)00216-2 8793038

[CR155] Ross DE (2011). Review of longitudinal studies of MRI brain volumetry in patients with traumatic brain injury. Brain Injury.

[CR156] Rostène W, Kitabgi P, Parsadaniantz SM (2007). Chemokines: a new class of neuromodulator?. Nature Reviews Neuroscience.

[CR157] Ruff RM, Young D, Gautille T, Marshall LF, Barth J, Jane JA (1991). Verbal learning deficits following severe head injury: heterogeneity in recovery over 1 year. Journal of Neurosurgery.

[CR158] Russo FA, Ives DT, Goy H, Pichora-Fuller MK, Patterson RD (2012). Age-related difference in melodic pitch perception is probably mediated by temporal processing: empirical and computational evidence. Ear and Hearing.

[CR159] Salthouse TA (2000). Aging and measures of processing speed. Biological Psychology.

[CR160] Salthouse TA (2010). Selective review of cognitive aging. Journal of the International Neuropsychological Society.

[CR161] Sander AM, Pappadis MR, Davis LC, Clark AN, Evans G, Struchen MA (2009). Relationship of race/ethnicity and income to community integration following traumatic brain injury: investigation in a non-rehabilitation trauma sample. NeuroRehabilitation.

[CR162] Scheff SW, Price DA, Hicks RR, Baldwin SA, Robinson S, Brackney C (2005). Synaptogenesis in the hippocampal CA1 field following traumatic brain injury. Journal of Neurotrauma.

[CR163] Schliebs R, Arendt T (2011). The cholinergic system in aging and neuronal degeneration. Behavioural Brain Research.

[CR164] Shors TJ, Anderson ML, Curlik DM, Nokia MS (2012). Use it or lose it: how neurogenesis keeps the brain fit for learning. Behavioural Brain Research.

[CR165] Sidaros A, Skimminge A, Liptrot MG, Sidaros K, Engberg AW, Herning M (2009). Long-term global and regional brain volume changes following severe traumatic brain injury: a longitudinal study with clinical correlates. NeuroImage.

[CR166] Simpson J, Kelly JP (2011). The impact of environmental enrichment in laboratory rats–behavioural and neurochemical aspects. Behavioural Brain Research.

[CR167] Sisti HM, Glass AL, Shors TJ (2007). Neurogenesis and the spacing effect: learning over time enhances memory and the survival of new neurons. Learning and Memory.

[CR168] Slovarp L, Azuma T, Lapointe L (2012). The effect of traumatic brain injury on sustained attention and working memory. Brain Injury.

[CR169] Small, B. J., Dixon, R. A., McArdle, J. J., Grimm, K. J. (2012). Do changes in lifestyle engagement moderate cognitive decline in normal aging? Evidence from the Victoria longitudinal study. *Neuropsychology, 26*(2), 144–155. 10.1037/a0026579 PMC3761970 22149165

[CR170] Smith C, Gentleman SM, Leclercq PD, Murray LS, Griffin WST, Graham DI (2013). The neuroinflammatory response in humans after traumatic brain injury. Neuropathology and Applied Neurobiology.

[CR171] Sozda CN, Larson MJ, Kaufman DA, Schmalfuss IM, Perlstein WM (2011). Error-related processing following severe traumatic brain injury: an event-related functional magnetic resonance imaging (fMRI) study. International Journal of Psychophysiology.

[CR172] Speisman RB, Kumar A, Rani A, Pastoriza JM, Severance JE, Foster TC (2013). Environmental enrichment restores neurogenesis and rapid acquisition in aged rats. Neurobiology of Aging.

[CR173] Störmer VS, Passow S, Biesenack J, Li SC (2012). Dopaminergic and cholinergic modulations of visual-spatial attention and working memory: insights from molecular genetic research and implications for adult cognitive development. Developmental Psychology.

[CR174] Struchen MA, Pappadis MR, Sander AM, Burrows CS, Myszka KA (2011). Examining the contribution of social communication abilities and affective/behavioral functioning to social integration outcomes for adults with traumatic brain injury. Journal of Head Trauma Rehabilitation.

[CR175] Taconnat L, Raz N, Toczé C, Bouazzaoui B, Sauzéon H, Fay S (2009). Ageing and organisation strategies in free recall: the role of cognitive flexibility. European Journal of Cognitive Psychology.

[CR176] Tate DF, York GE, Reid MW, Cooper DB, Jones L, Robin DA (2014). Preliminary findings of cortical thickness abnormalities in blast injured service members and their relationship to clinical findings. Brain Imaging and Behavior.

[CR177] Till C, Colella B, Verwegen J, Green RE (2008). Postrecovery cognitive decline in adults with traumatic brain injury. Archives of Physical Medicine and Rehabilitation.

[CR178] Toni N, Teng EM, Bushong EA, Aimone JB, Zhao C, Consiglio A (2007). Synapse formation on neurons born in the adult hippocampus. Nature Neuroscience.

[CR179] Torasdotter M, Metsis M, Henriksson BG, Winblad B, Mohammed AH (1996). Expression of neurotrophin-3 mRNA in the rat visual cortex and hippocampus is influenced by environmental conditions. Neuroscience Letters.

[CR180] Torasdotter M, Metsis M, Henriksson BG, Winblad B, Mohammed AH (1998). Environmental enrichment results in higher levels of nerve growth factor mRNA in the rat visual cortex and hippocampus. Behavioural Brain Research.

[CR181] Truong JQ, Ciuffreda KJ, Han MHE, Suchoff IB (2014). Photosensitivity in mild traumatic brain injury (mTBI): a retrospective analysis. Brain Injury.

[CR182] Turner B, Fleming J, Cornwell P, Worrall L, Ownsworth T, Haines T (2007). A qualitative study of the transition from hospital to home for individuals with acquired brain injury and their family caregivers. Brain Injury.

[CR183] Turner B, Ownsworth T, Cornwell P, Fleming J (2009). Reengagement in meaningful occupations during the transition from hospital to home for people with acquired brain injury and their family caregivers. American Journal of Occupational Therapy.

[CR184] Turner GR, McIntosh AR, Levine B (2011). Prefrontal compensatory engagement in TBI is due to altered functional engagement of existing networks and not functional reorganization. Frontiers in Systems Neuroscience.

[CR185] Vance DE, Roberson AJ, McGuinness TM, Fazeli PL (2010). How neuroplasticity and cognitive reserve protect cognitive functioning. Journal of Psychosocial Nursing and Mental Health Services.

[CR186] Van Praag, H., Kempermann, G. and Gage, F. (2000). Neural consequences of environmental enrichment. *Neuroscience, 1*, 191–198. 10.1038/35044558 11257907

[CR187] Waddell J, Shors TJ (2008). Neurogenesis, learning and associative strength. European Journal of Neuroscience.

[CR188] Wallace CS, Withers GS, Farnand A, Lobingier BT, McCleery EJ (2011). Evidence that angiogenesis lags behind neuron and astrocyte growth in experience-dependent plasticity. Developmental Psychobiology.

[CR189] Wang HX, Jin Y, Hendrie HC, Liang C, Yang L, Cheng Y (2013). Late life leisure activities and risk of cognitive decline. Journals of Gerontology Series A: Biological Sciences and Medical Sciences.

[CR190] Weatherbee SR, Gamaldo AA, Allaire JC (2009). Exploring the within-person coupling of reading vision and cognition in the elderly. Neuropsychology, Development, and Cognition. Section B, Aging, Neuropsychology and Cognition.

[CR191] Wheaton P, Mathias JL, Vink R (2009). Impact of early pharmacological treatment on cognitive and behavioral outcome after traumatic brain injury in adults: a meta-analysis. Journal of Clinical Psychopharmacology.

[CR192] Willemse-van Son AH, Ribbers GM, Hop WC, Stam HJ (2009). Community integration following moderate to severe traumatic brain injury: a longitudinal investigation. Journal of Rehabilitation Medicine.

[CR193] Winocur G (1998). Environmental influences on cognitive decline in aged rats. Neurobiology of Aging.

[CR194] Winocur G, Moscovitch M (1990). A comparison of cognitive function in community-dwelling and institutionalized old people of normal intelligence. Canadian Journal of Psychology.

[CR195] Wise EK, Mathews-Dalton C, Dikmen S, Temkin N, Machamer J, Bell K (2010). Impact of traumatic brain injury on participation in leisure activities. Archives of Physical Medicine and Rehabilitation.

[CR196] Wood RL, McHugh L (2013). Decision making after traumatic brain injury: a temporal discounting paradigm. Journal of the International Neuropsychological Society.

[CR197] World Health Organization (2013). *How to use the ICF: a practical manual for using the International Classification of Functioning, Disability, and Health (ICF), exposure draft for comment*. Retrieved from http://www.who.int/classifications/drafticfpracticalmanual2.pdf?ua=1.

[CR198] Writer BW, Schillerstrom JE (2009). Psychopharmacological treatment for cognitive impairment in survivors of traumatic brain injury: a critical review. Journal of Neuropsychiatry and Clinical Neurosciences.

[CR199] Yoon C, Cole CA, Lee MP (2009). Consumer decision making and aging: current knowledge and future directions. Journal of Consumer Psychology.

[CR200] Young-Bernier M, Kamil Y, Tremblay F, Davidson PSR (2012). Associations between a neurophysiological marker of central cholinergic activity and cognitive functions in young and older adults. Behavioral and Brain Functions.

